# Characterizing the Role of HMG-CoA Reductase in Aryl Hydrocarbon Receptor-Mediated Liver Injury in C57BL/6 Mice

**DOI:** 10.1038/s41598-019-52001-2

**Published:** 2019-11-01

**Authors:** Peter Dornbos, Amanda Jurgelewicz, Kelly A. Fader, Kurt Williams, Timothy R. Zacharewski, John J. LaPres

**Affiliations:** 1 Department of Biochemistry and Molecular Biology, Michigan State University, East Lansing, MI 48824 USA; 2 Institute for Integrative Toxicology, Michigan State University, East Lansing, MI 48824 USA; 3 Department of Pharmacology and Toxicology, Michigan State, East Lansing, MI 48824 USA; 4 Department of Pathobiology and Diagnostic Investigation, Michigan State, East Lansing, MI 48824 USA

**Keywords:** Biochemistry, Molecular biology

## Abstract

The aryl hydrocarbon receptor (AHR) is a ligand-activated transcription factor. The prototypical ligand of the AHR is an environmental contaminant called 2,3,7,8-tetrachlorodibenzo-*p*-dioxin (TCDD). TCDD exposure is associated with many adverse health outcomes in humans including non-alcoholic fatty liver disease (NAFLD). Previous studies suggest that AHR ligands alter cholesterol homeostasis in mice through repression of genes involved in cholesterol biosynthesis, such as *Hmgcr*, which encodes the rate-limiting enzyme of cholesterol biosynthesis called 3-hydroxy-3-methyl-glutaryl coenzyme A reductase (HMGCR). In this study, we sought to characterize the impact of HMGCR repression in TCDD-induced liver injury. C57BL/6 mice were exposed to TCDD in the presence or absence of simvastatin, a competitive inhibitor of HMGCR. Simvastatin exposure decreased TCDD-induced hepatic lipid accumulation in both sexes, but was most prominent in females. Simvastatin and TCDD (S + T) co-treatment increased hepatic AHR-battery gene expression and liver injury in male, but not female, mice. In addition, the S + T co-treatment led to an increase in hepatic glycogen content that coincides with heavier liver in female mice. Results from this study suggest that statins, which are amongst the most prescribed pharmaceuticals, may protect from AHR-mediated steatosis, but alter glycogen metabolism and increase the risk of TCDD-elicited liver damage in a sex-specific manner.

## Introduction

The aryl hydrocarbon receptor (AHR) is a ligand-activated transcription factor that primarily resides in the cytoplasm prior to activation. While endogenous ligands of the AHR have been identified^1–4^, the AHR has a high-affinity for an environmental contaminant called 2,3,7,8-tetrachlorodibenzo-*p*-dioxin (TCDD)^5^. The canonical AHR signaling pathway has been well-characterized. Once activated, the AHR translocates to the nucleus and forms a heterodimer with the aryl hydrocarbon receptor nuclear translocator (ARNT)^6–8^. The AHR:ARNT heterodimer binds to dioxin response elements (DREs) containing a core sequence of 5′-GCGTG-3′^9,10^. Previous reports have identified DREs located throughout the genome and that the AHR pathway mediates the differential expression a diverse set of genes^11^.

In humans, TCDD exposure is associated with a plethora of adverse health outcomes, such as chloracne, immunosuppression, and cancer^12–15^. Accumulating evidence suggests that TCDD and other dioxin-like chemicals are also associated with increased incidence of metabolic disorders such as nonalcoholic fatty-liver disease (NAFLD)^16,17^. In rodents, TCDD treatment leads to accumulation of lipids in the liver that can progress to steatohepatitis with fibrosis^18–21^. Much remains unknown about the link between AHR-elicited gene expression and the onset and progression of NAFLD.

Rodent-based studies have indicated that AHR regulates cholesterol biosynthesis. AHR ligands suppress several key genes involved in cholesterol synthesis in mice and human hepatocytes, including the gene that encodes the rate-limiting enzyme, 3-hydroxyl-3-methylglutaryl-CoA reductase (HMGCR)^22^. TCDD exposure has been associated with marked reductions in circulating total cholesterol (TC), high-density lipoprotein (HDL), and low-density lipoprotein (LDL) in mice^23^. Further studies have shown that, while hepatic cholesterol biosynthesis is repressed, repeated TCDD treatment leads to increased hepatic cholesterol ester (CE) content in mice which is possibly due to repression of VLDL secretion and decreased bile acid synthesis and secretion^18,24^.

Given the complex regulation of cholesterol biosynthesis and its importance to organismal health, we sought to better-characterize the impact of HMGCR repression in TCDD-induced liver injury. Male and female C57BL/6 mice were co-treated with TCDD and simvastatin (S + T), a competitive inhibitor of HMGCR. Our results indicate that HMGCR activity likely plays a key, but sex-specific, role in TCDD-elicited liver injury. While TCDD-mediated lipid deposition in the liver was decreased in S + T co-treated male and female mice, males were found to have greater degree of liver damage and females were found to have increased levels of hepatic glycogen. As such, this study suggests that statins used to control hypercholesterolemia may increase the risk of sex-specific liver injury from exposure to AHR ligands, such as TCDD.

## Methods

### Statin and TCDD mouse exposure

All animal experiments were approved by Michigan State University’s Institutional Animal Care & Use Committee and were carried out in accordance with this approval and all relevant guidelines and regulations. Age-matched male and female C57BL/6 mice were ordered from Charles River Laboratories (Kingston, NY) and delivered to Michigan State University on postnatal day 25 (PND25). Mice were acclimated to the facility for 7 days prior to treatment. Mice were housed in Innovive Innocages (Innovive, San Diego, CA) with ALPHA-Dri bedding (Shepherd Specialty Papers, Chicago, IL) under constant 12-hour light/dark cycles, temperature, and humidity. Upon arrival, mice were randomly placed into a treatment group: (a) sesame oil (vehicle control; Sigma Aldrich) + standard mouse chow (Harlan Teklad Rodent Diet 8940), (b) TCDD (10 µg/kg; AccuStandard, New Haven, CT) + standard mouse chow, c) sesame oil + chow containing simvastatin (500 mg/kg food, Harlan Teklad Rodent Diet 8940; Sigma Aldrich, St. Louis, MO), or d) TCDD + chow containing simvastatin. The simvastatin-laced chow was prepared at Envigo (Huntingdon, UK). Mice were acclimated to the simvastatin-laced chow for 3 days prior to treatment with TCDD. Chow was provided *ad libitum* for each treatment group. Each treatment group had a sample size of 8 mice with the exception of the male TCDD + standard chow group which had 7 mice; notably, the aforementioned group with a smaller sample size was chosen at random and was due to mouse dying in-transit prior to the experiment start-point. The average (mg/kg body weight/day) and standard deviation of simvastatin exposure for females and males over the 13-day period was 77.2 ± 2.8 and 73.6 ± 1.2, respectively. TCDD treatment did not significantly impact consumption of simvastatin-laced chow. Following the dosing regime, the mice were sacrificed on day 11 following a 6 hour fasting-period. Tissues were either frozen in liquid nitrogen or fixed in 10% phosphate-buffered formalin (Thermo Fisher, Waltham, MA).

### Histological analyses

Formalin-fixed liver was vacuum infiltrated with paraffin using a Tissue-Tek VIP 2000 and embedded with the HistoCentre III embedding station (Thermo Fisher, Waltham, MA). A Rechert Jung 2030 rotary microtome (Reichert, Depew NY) was used to section tissue at 4–5 µm. Sections were then placed on slides and dried for 2–24 h at 56 °C. Dried liver sections were stained with hematoxylin and eosin (H&E) for general morphometric analysis and with periodic acid–Schiff (PAS) to detect glycogen. Histological severity scoring of H&E stained liver sections was performed by a certified pathologist and based on the following scale: 0 = no lesions present; 1 = mild and random foci of inflammation; 2 = intermediate inflammation with presence of necrotic hepatocytes; and 3 = marked inflammation and greater presence of necrotic hepatocytes as compared to other histologic scores. In all cases, n ≥ 7 for each dose group during the histologic scoring. Frozen tissues were sectioned at 6 µm and stained with oil red O (ORO) to detect neutral lipids as previously described^25^. An Olympus Virtual Slide System VS110 was used to digitize the slides at 20x magnification (Olympus, Center Valley, PA). The Olympus OlyVIA software (Olympus) was used to visualize the digitized slides. The percent area of liver tissue stained with ORO was quantified using the Quantitation Histological Analysis Tool (QuHAnT) as previously described^26^. The optimal hue, saturation, and value (HSV) thresholds used for feature extraction were 0 to 50 and 225 to 250 (hue), 30 to 255 (saturation), and 0 to 255 (value), while the optimal total tissue feature extraction thresholds were 0 to 255 (hue), 20 to 255 (saturation), and 0 to 255 (value). All histological processing and staining was performed by the Michigan State University Investigative Histopathology Laboratory.

### Hepatic gene expression

Frozen liver was homogenized in 1 mL of TRIzoL reagent using a Mixer Mill 300 (Life Sciences, Carlsbad, CA). RNA was extracted using an additional 5:1 phenol:chloroform step (Sigma Aldrich, St. Louis, MO). The quantity and purity (260/280 ratio) of RNA was analyzed with a NanoDrop 1000. Total RNA (2 µg) was converted to cDNA using oligo(dT) primers and reverse transcriptase superscript III. SYBR green Mastermix (Life Technologies) was used to analyze relative gene expression. Gene expression was normalized to the geometric mean of three house-keeping genes: (1) *Hprt*, (2) *Actb*, and (3) *Gusb*. Primer sequences are listed in Table S1. All PCR was performed using either a DNA Engine Opticon 2 (Bio-Rad, Hercules CA) or a QuantStudio 7 Flex Real-Time PCR System (Thermo Fisher, Waltham, MA). The 2^−∆∆CT^ method was used to calculate fold changes and all values are relative to the mean of vehicle control mice on standard diet. In all cases, sample size (n) is ≥7 in all groups.

### Western blot analysis

Frozen liver was homogenized in radioimmunoprecipitation assay (RIPA) buffer using a Mixer Mill 300 (Life Sciences, Carlsbad, CA). The protein concentration in the supernatant was determined with a Bradford Assay following a 10 min centrifugation (16,000 × g)^27^. Sodium dodecyl sulfate-polyacrylamide gel electrophoresis (SDS-PAGE) was used to separate 15 µg of total protein which was subsequently transferred to a nitrocellulose membrane. The membrane was blocked with 5% non-fat dry milk dissolved in Tris-Buffered Saline with 0.05% tween 20 (TBST) and probed with monoclonal anti-mouse HMGCR antibody (1:1000; Abcam, Cambridge, MA) or a monoclonal beta-actin (ACTB) antibody (1:3000; Santa Cruz Biotechnology, Dallas, TX) overnight at 4**°**C. Following 3 × 5-minute washes with TBST, the membrane was exposed to a monoclonal mouse anti-rabbit IgG-HRP (1:1000; Santa Cruz Biotechnology, Dallas, TX) or a mouse IgG kappa binding protein-HRP (1:3000; Santa Cruz Biotechnology, Dallas, TX) where appropriate. Following 3 × 5-minute washes with TBST, the blots were developed using the Pierce enhanced chemiluminescence (ECL) Western Blotting Substrate (Thermo Fisher, Waltham, MA). The Image Studio Lite software (LI-COR, Lincoln, NE) was used for the densitometry analysis. HMGCR expression was normalized to ACTB prior to analysis (Table S2). Fold changes are relative to the mean of vehicle control mice on standard diet. The sample size (n) was 5 for each group and mice were randomly selected.

### Serum clinical chemistry

Serum total cholesterol, low-density lipoprotein (LDL), alanine aminotransferase (ALT) and glucose levels were measured using commercially-available reagents (FUJIFILM Wako Diagnostics, Richmond, VA). Serum triglycerides were measured with commercially-available reagents (Pointe Scientific, Canton, MI). Serum high-density lipoprotein (HDL) was quantified with a commercially-available kit (Crystal Chemical, Houston, TX). Serum free fatty acids and ketone bodies (i.e. beta hydroxybutyrate) were measured using commercially-available kits (Cayman Chemical, Ann Arbor, MI). In all cases, a SpectraMax M2 microplate reader was used (Molecular Devices, San Jose, CA). The sample size (n) was ≥ 5 for each group; mice were randomly selected.

### Hepatic lipid extraction

Hepatic lipids were extracted as previously described^28^. Frozen liver was homogenized in 10x volume of extraction buffer (18 mM Tris (pH 7.5), 300 mM D-Mannitol, 50 mM EGTA, and 0.1 mM phenylmethylsulfonyl fluoride) using a Mixer Mill 300 (Life Sciences, Carlsbad, CA). 500 µL of homogenate was added to 4 mL of 2:1 chloroform:methanol and mixed end-over-end shaking overnight at room temperature. H_2_0 (800 μL) was then added and the sample was mixed by vortexing and the phases were separated by centrifugation (3000 × g for 5 min). 2 mL of the organic phase was transferred to a new tube and evaporated over nitrogen to dryness. Following an incubation at 45 °C for 5 min, the lipid residue was dissolved in 300 uL of isopropyl alcohol with 10% Triton X-100. Commercially-available reagents were used to analyze triglycerides (Pointe Scientific, Canton, MI) and total cholesterol (FUJIFILM Wako Diagnostics, Richmond, VA) with a SpectraMax M2 microplate reader (Molecular Devices, San Jose, CA). The sample size (n) was 5 for each group and mice were randomly selected.

### Hepatic glycogen and glucose assay

The level of glycogen and free glucose were determined as previously described^20^. Frozen liver (~50 mg) was homogenized in 250 µL of 6% perchloric acid using a Polytron PT21000 (Kinematica AG, Luzern, Switzerland). A portion of the homogenate was used to measure background glucose while another portion of the homogenate (50 µL) was combined with 25 µL of 1 M NaHCO_3_ and 125 µL of amyloglucosidase solution (2 mg/mL; Sigma Aldrich, St. Louis, MO). The mixtures were incubated with shaking at 37 °C for 2 hours. Background-corrected glucose levels in the amyloglucosidase-treated samples were used to infer hepatic glycogen levels. Glucose was assessed using commercially-available reagents (FUJIFILM Wako Diagnostics, Richmond, VA) and a SpectraMax M2 microplate reader (Molecular Devices, San Jose, CA). The sample size (n) was 5 for each group and mice were randomly selected.

### Statistical analyses

All statistical analyses were performed using version 3.0.2 in R^29^. The normality of data distributions was analyzed with histograms and q-q plots prior to downstream statistical analyses. Outliers within dose-groups were assessed via the Grubbs’ test and removed if significant (p ≤ 0.05). Differences across treatment groups were assessed with a one-way analysis of variance (ANOVA) with a Tukey’s pair-wise posthoc test with a p ≤ 0.05 considered significant.

## Results

### Characterizing hepatic expression of *Hmgcr*

**Figure 1:**
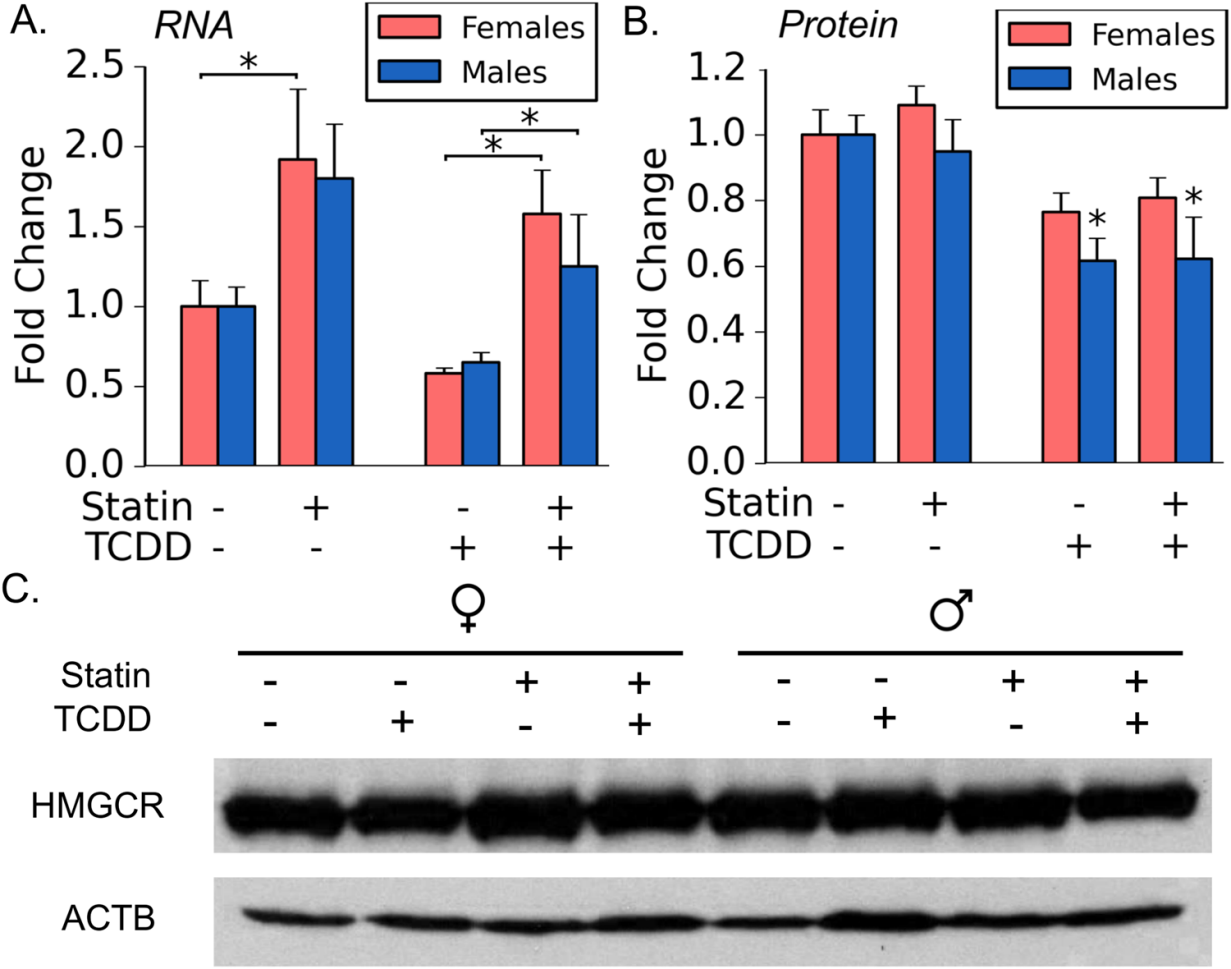
Simvastatin and TCDD-elicited effects of the expression of HMG-CoA reductase. Expression of *Hmgcr* was analyzed by QRTPCR for females and males (**A**). For QRTPCR analysis, all samples are reported as fold changes which are relative to vehicle control (i.e. sesame oil); in all cases, sample sizes (n) were ≥7. Densitometry analysis of western blots was used to assess relative protein expression of HMGCR for females and males (**B**). Densitometry analysis is reported as fold changes relative to vehicle control (i.e. sesame oil); in all cases, sample sizes (n) were 5. Asterisks (*) indicate statistically significant differences (p ≤ 0.05) as compared to the respective vehicle control (i.e. sesame oil vs. TCDD treatment or simvastatin-treatment vs. simvastatin + TCDD co-treatment) or between means indicated by brackets. A representative western blot was chosen to visualize the bands used for densitometry analysis; the bands pictured are from the same gel and same exposure time of 1 minute (**C**). The original, uncropped gel image can be found in the Supplemental Material (Fig. S1).

To characterize the potential role of HMGCR activity in TCDD-elicited toxicity, C57BL/6 mice were treated with either sesame oil (i.e. vehicle) or TCDD (10 µg/kg/day) for 10 consecutive days in the presence of absence of chow containing simvastatin (500 mg/kg chow), a competitive inhibitor of HMGCR. In analyzing gene expression levels of *Hmgcr*, TCDD exposure reduced hepatic *Hmgcr* mRNA levels for both male and female mice fed either standard or simvastatin-laced chow, but not in a statistically significant manner (Fig. 1A). In female mice, both simvastatin alone and the simvastatin + TCDD co-treatment (S + T) significantly increased hepatic *Hmgcr* mRNA expression as compared to vehicle or TCDD-treatment, respectively (Fig. 1A). In male mice, *Hmgcr* mRNA levels were significantly higher in the S + T group as compared to TCDD alone, but, unlike females, simvastatin alone did not significantly increase expression of *Hmgcr*.

Expression of several other key genes were analyzed to investigate the impact of simvastatin on cholesterol biosynthesis beyond *Hmgc*r, such as 7-dehydrocholesterol reductase (*Dhcr7*), squalene epoxidase (*Sqle*), and Cytochrome P450, family 51 (*Cyp51*) (Table 1). Overall, the expression of these genes follow very similar patterns as was observed with *Hmgcr* for mice of both sexes. TCDD treatment in both sexes led to an overall reduction in expression of cholesterol synthesis-related genes; the differences, however, were not statistically significant. Similarly, simvastatin exposure alone in both sexes led to an overall increase in expression of genes involved in cholesterol biosynthesis. Notably, increases in *Sqle* and *Cyp51* were statistically significant for females while only increases in Sqle expression were significant for males. Furthermore, mice of both sexes that were co-treated with S + T were observed to have an overall increased level of expression in genes involved in cholesterol biosynthesis as compared to TCDD alone; expression of *Dhcr7*, *Sqle*, and *Cyp51* were significantly increased as compared to TCDD alone in females while *Dhcr7* and *Sqle* was significantly increased in males.

**Table 1:** Simvastatin and TCDD-mediated changes in gene expression.

Gene	Females				Males			
	Standard Chow		Statin Chow		Standard Chow		Statin Chow	
	Sesame Oil	TCDD	Sesame Oil	TCDD	Sesame Oil	TCDD	Sesame Oil	TCDD
*Apoa1*	1.00 (0.11)	0.26 (0.02)^a^	0.97 (0.12)	0.22 (0.02)^c^	1.00 (0.14)	0.33 (0.05)^a^	1.93 (0.05)^b^	0.36 (0.07)^c^
*Cyp1a1*	1.00 (0.26)	2959.93 (104.63)^a^	1.08 (0.86)	2625.40 (315.80)^c^	1.00 (0.13)	2514.14 (343.20)^a^	1.46 (0.15)	4070.06 (405.41)^b,c^
*Cyp1a2*	1.00 (0.04)	16.51 (1.13)^a^	0.76 (0.11)	14.44 (1.19)^c^	1.00 (0.06)	12.85 (2.00)^a^	0.98 (0.06)	17.56 (1.99)^b,c^
*Cyp1b1*	1.00 (0.21)	505.86 (60.38)^a^	0.81 (0.19)	377 (20.43)^c^	1.00 (0.10)	701.56 (95.72)^a^	0.99 (0.09)	1320.70 (167.30)^b,c^
*Cyp4a10*	1.00 (0.15)	0.36 (0.04)^a^	0.51 (0.08)^b^	0.19 (0.03)^b,c^	1.00 (0.22)	0.32 (0.06)^a^	1.11 (0.14)	0.78 (0.14)^b^
*Cyp4a14*	1.00 (0.24)	0.57 (0.08)^a^	0.71 (0.12)	0.27 (0.03)^b,c^	1.00 (0.29)	0.11 (0.02)^a^	1.4 (0.20)	0.35 (0.08)^b,c^
*Cyp51*	1.00 (0.23)	0.49 (0.03)	2.12 (0.15)^a^	1.59 (0.21)^b^	1.00 (0.11)	0.56 (0.19)	1.86 (0.23)	1.01 (0.19)
*Dhcr7*	1.00 (0.26)	0.55 (0.10)	1.63 (0.17)	1.46 (0.21)^b^	1.00 (0.12)	0.64 (0.18)	1.67 (0.17)	1.00 (0.21)^b^
*Gbe1*	1.00 (0.11)	0.51 (0.04)^a^	0.60 (0.04)^a^	0.48 (0.06)	1.00 (0.13)	0.42 (0.06)^a^	0.82 (0.12)	0.63 (0.08)
*Gys2*	1.00 (0.15)	0.49 (0.04)^a^	0.78 (0.10)	0.28 (0.03)^b,c^	1.00 (0.18)	0.24 (0.04)^a^	1.05 (0.16)	0.36 (0.08)^c^
*Hk1*	1.00 (0.07)	1.10 (0.06)	0.66 (0.02)^a^	1.04 (0.05)^c^	1.00 (0.08)	1.45 (0.15)	1.03 (0.24)	1.56 (0.12)
*Lcat*	1.00 (0.31)	0.70 (0.05)	0.63 (0.04)	0.57 (0.06)	1.00 (0.11)	1.19 (0.14)	1.42 (0.14)	1.48 (0.24)
*Ldlr*	1.00 (0.11)	0.87 (0.05)	0.97 (0.11)	1.26 (0.14)	1.00 (0.15)	0.75 (0.06)	0.95 (0.10)	0.92 (0.23)
*Pgm1*	1.00 (0.15)	0.99 (0.05)	0.69 (0.04)^a^	0.93 (0.08)	1.00 (0.10)	1.17 (0.20)	0.98 (0.12)	1.50 (0.15)
*Ppara*	1.00 (0.20)	0.54 (0.05)^a^	0.63 (0.08)	0.32 (0.05)^b,c^	1.00 (0.16)	0.44 (0.04)^a^	0.96 (0.11)	0.85 (0.10)^b^
*Pygl*	1.00 (0.14)	0.32 (0.03)^a^	0.53 (0.05)^b^	0.24 (0.02)^c^	1.00 (0.11)	0.37 (0.10)^a^	1.01 (0.15)	0.39 (0.05)^c^
*Sqle*	1.00 (0.45)	0.43 (0.06)	2.95 (0.23)^a^	3.93 (0.38)^b^	1.00 (0.12)	0.78 (0.11)	2.09 (0.27)^a^	1.41 (0.20)^b^
*Ugp2*	1.00 (0.14)	0.42 (0.03)^a^	0.71 (0.05)	0.39 (0.04)^c^	1.00 (0.15)	0.28 (0.07)^a^	0.80 (0.11)	0.30 (0.03)^c^

Results are presented as fold changes with standard error in parentheses which are relative to vehicle control mice. In all cases, sample size (n) ≥7 in all groups for gene expression analysis. Superscript letters indicate significant differences (p < 0.05) as indicated by an ANOVA in comparison to: ^a^vehicle (sesame oil), ^b^TCDD treatment, or ^c^simvastatin treatment. Statistical comparisons were not made across sexes.

Given the focus on *Hmgcr* in this study, we also analyzed expression of HMGCR at the protein level. Western blot analysis suggested that TCDD exposure significantly repressed HMGCR in the liver of male mice regardless of diet (Fig. 1B,C). While we observed a similar decrease in female hepatic HMGCR expression, the difference was not significant as compared to control mice. To the contrary with the gene expression results, protein-level analysis suggests that simvastatin alone or the S + T co-treatment did not mediate an increase in expression of hepatic HMGCR expression as compared to control mice in either male or female mice (Fig. 1B,C).

### Assessing serum and hepatic cholesterol levels

The levels of total cholesterol (TC), low-density lipoprotein (LDL), and high-density lipoprotein (HDL) cholesterol in the serum were quantified to characterize the impact of the differing treatments on cholesterol homeostasis (Table 2). In comparing TC levels, male and female mice that were treated with TCDD alone or co-treated with S + T had significantly less TC in serum as compared to control mice and mice treated with simvastatin alone. There were no differences in TC levels in comparing S + T co-treated and TCDD treated mice. Serum LDL levels in male mice that were co-treated with S + T were significantly lower as compared to those treated with simvastatin alone. LDL levels were not impacted by any treatment in female mice. Along these lines, hepatic gene expression of the LDL receptor (*Ldlr*) was not altered by any treatments in males or females (Table 1). The most dramatic changes were found in the serum HDL levels. TCDD treatment alone led to significant reductions in HDL levels in mice of both sexes (Table 2). Interestingly, simvastatin treatment alone led to a significant decrease the level of HDL in females, but not in male mice. As such, we observed the HDL:LDL ratio in the standard chow fed females as nearly double the HDL:LDL ratio in simvastatin-treated females (~2,1 vs ~1.1). In comparison, the HDL:LDL ratios were not altered when comparing standard chow and simvastatin-treated males. To assess a potential mechanism to the sex difference, we analyzed hepatic gene expression of the scavenger receptor class B type 1 gene (*Scarb1*) which serves as the HDL receptor. *Scarb1* expression was not altered in the liver by any treatment in females (Table 1). While serum HDL levels were lower in TCDD-treated males regardless of diet (Table 1), *Scarb1* expression was significantly repressed in males treated with TCDD alone as compared to controls, but not different in comparing the S + T co-treatment and TCDD-treatment groups (Table 1). Expression of the apolipoprotein gene A1 (*Apoa1*) was significantly repressed by TCDD in males and females regardless of simvastatin treatment (Table 1). In contrast, *Apoa1* was found to significantly induced in simvastatin-treated male mice, but was not found different in simvastatin-treated female mice. Expression of lecithin cholesterol acyltransferase (*Lcat*), which is involved in maturation of HDL, was found to be lower and trending towards statistical significance in the female mice treated with simvastatin alone (p = 0.06; Table 1). To the contrary, *Lcat* expression was not lower in male mice treated with simvastatin alone, but rather was slightly increased.

**Table 2:** Cholesterol levels, quantitative pathology, and serum clinical chemistry data for TCDD and simvastatin co-treatment study.

Group	Measurement	Females				Males			
		Standard Chow		Statin Chow		Standard Chow		Statin Chow	
		Sesame Oil	TCDD	Sesame Oil	TCDD	Sesame Oil	TCDD	Sesame Oil	TCDD
**Cholesterol**	Low-Density Lipoprotein (mg/dL)	38.1 (1.5)	34.8 (1.0)	52.7 (3.2)	35.6 (1.0)	40.4 (0.7)	33.1 (0.5)	43.2 (0.8)	29.3 (1.6)^c^
	High-Density Lipoprotein (mg/dL)	80.0 (3.8)	43.7 (1.5)^a^	58.1 (2.1)^a^	37.9 (0.9)^c^	63.2 (2.3)	35.1 (1.2)^a^	70.2 (2.1)	39.1 (2.5)^c^
	Total Cholesterol (mg/dL)	136.7 (1.9)	120.4 (1.5)^a^	137.3 (1.3)	120.5 (0.9)^c^	151.7 (1.5)	122.9 (1.7)^a^	170.0 (1.0)	133.6 (2.7)^c^
	Hepatic Free Cholesterol (mg/g)	2.3 (0.1)	3.9 (0.1)^a^	2.2 (0.1)	3.2 (0.2)^b,c^	2.1 (0.1)	3.2 (0.1)^a^	2.1 (0.1)	2.8 (0.1)^b,c^
**Pathology**	Liver Weight (g)	0.76 (0.02)	0.99 (0.02)^a^	0.80 (0.02)	1.12 (0.04)^b,c^	1.13 (0.04)	1.27 (0.07)	1.18 (0.03)	1.31 (0.05)
	Normalized Liver Weight (mg/kg)	48.48 (0.86)	65.89 (0.63)^a^	50.83 (0.45)	73.47 (1.83)^b,c^	58.58 (1.39)	71.27 (2.67)^a^	59.38 (1.33)	73.80 (1.69)^c^
	Body Weight (g)	15.6 (0.3)	15.1 (0.3)	15.7 (0.3)	15.2 (0.3)	19.5 (0.3)	17.8 (0.4)^a^	19.9 (0.2)	17.7 (0.5)^c^
	GWAT Weight (g)	0.12 (0.02)	0.1 (0.01)	0.10 (0.009)	0.10 (0.01)	0.23 (0.01)	0.22 (0.01)	0.25 (0.005)	0.22 (0.01)
	Normalized GWAT Weight (mg/kg)	7.7 (1.39)	7.09 (0.57)	6.17 (0.60)	6.85 (2.08)	11.79 (0.76)	12.39 (0.24)	12.39 (0.24)	12.65 (0.42)
	Average Histologic Severity Score	0.8 (0.2)	2.0 (0.0)^a^	0.4 (0.2)	1.9 (0.1)^c^	0.4 (0.2)	2.6 (0.2)^a^	0.3 (0.2)	1.5 (0.3)^b,c^
**Clinical Chemistry**	Alanine Aminotransferase (mg/dL)	44.3 (2.2)	335.1 (21.0)^a^	34.6 (0.6)	345.6 (19.5)^c^	34.0 (1.4)	1844.5 (91.5)^a^	94.2 (3.1)	3010.9 (256.9)^b,c^
	Free Fatty Acids (mmol/mL)	662.7 (19.5)	591.0 (14.4)	470.3 (26.2)	266.6 (25.6)^b,c^	437.4 (8.1)	472.9 (33.2)	325.4 (20.6)	397.0 (32.4)
	Ketone Bodies (mg/dL)	300.6 (11.3)	208.2 (11.2)	263.7 (11.6)	72.2 (2.0)^b,c^	85.6 (3.2)	145.5 (3.8)^a^	83.6 (2.4)	130.0 (5.1)^c^
	Glucose (mg/dL)	165.4 (2.2)	153.7 (3.9)	165.2 (2.4)	142.0 (3.3)	223.7 (4.1)	158.8 (2.7)^a^	221.9 (3.4)	156.6 (3.0)^c^
	Triglycerides (mg/dL)	99.9 (1.3)	107.5 (0.7)	99.0 (1.2)	104.7 (1.8)	115.6 (1.5)	108.4 (1.2)	108.4 (1.3)	101.5 (1.3)

Results are presented as mean weights with standard error in parenthesis. In all cases, sample size (n) is ≥5 in all groups. Superscript letters indicate significant differences (p < 0.05) as indicated by an ANOVA in comparison to: ^a^vehicle (sesame oil), ^b^TCDD treatment, or ^c^simvastatin treatment. Statistical comparisons were not made across sexes.

Hepatic total cholesterol levels were also analyzed to characterize the impact of each treatment on the hepatic cholesterol flux (Table 1). Male and female mice displayed similar trends in comparing hepatic cholesterol levels across treatment groups. TCDD treatment alone led to significantly higher levels of hepatic cholesterol as compared to control mice in males and females. Similarly, S + T co-treatment led to significantly greater levels of hepatic cholesterol as compared to control and simvastatin treated mice in both sexes. We also observed that, in both sexes, the S + T co-treated mice had significantly less hepatic cholesterol as compared to TCDD-treated mice.

### Impacts on gross and hepatic pathology

Of particular note was a discrepancy between sexes when comparing liver weights of TCDD treated and S + T co-treated mice. In assessing gross pathology, female mice that were treated with TCDD and co-treated with S + T led to significantly higher total liver weight (TLW) and body-weight normalized liver weights (NLW) as compared to the respective controls (Table 2). In males, TLW was not affected, but TCDD and S + T co-treatment led to significantly-heavier NLWs. Moreover, in females, the S + T treatment led to significantly-heavier TLWs and NLWs as compared to mice treated with TCDD alone. In male mice, liver weights were not different in comparing S+T to TCDD treated mice.

**Figure 2:**
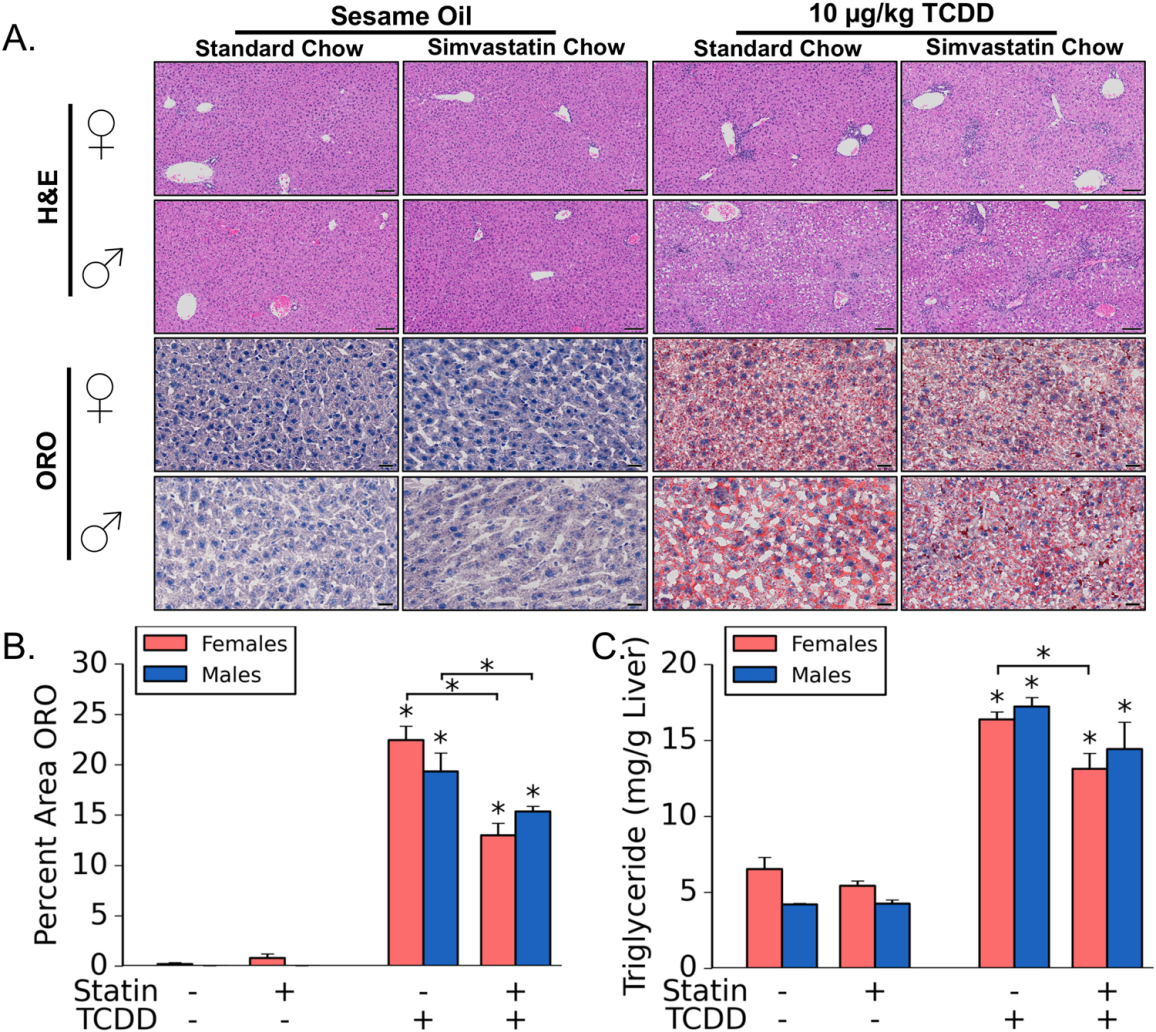
Impact of simvastatin and TCDD co-exposure on hepatic lipid accumulation. Hematoxylin and eosin (H&E) staining of liver was used to assess general morphology and oil red O (ORO) staining was used to assess neutral lipid. Representative samples for each stain were chosen for each treatment group (**A**). Scale bars represents 100 µm for H&E and 50 µm for ORO. Percent area of tissue stained with ORO was quantified with QuHAnT software (**B**). Triglycerides levels in hepatic lipid extracts were quantified with commercially-available reagents (**C**). In all cases, sample sizes (n) were ≥5. Asterisks (*) over bars indicate statistically significant differences (p ≤ 0.05) as compared to the respective vehicle control (i.e. sesame oil vs. TCDD treatment or simvastatin-treatment vs. simvastatin + TCDD co-treatment) or between means indicated by brackets.

Tissue sections were stained with hematoxylin and eosin (H&E) to assess general morphology and to perform severity scoring (See Methods; Fig. 2A, Table 2). TCDD exposure alone led to a significantly greater level of inflammation, hepatocyte necrosis, and increases in vacuolization in both sexes. TCDD exposed mice were also found to have increased levels of alanine aminotransferase (ALT) levels in serum (Table 2). There were two primary histological discrepancies in comparing TCDD treatment- and S + T co-treatment-induced liver pathology across sexes. S + T co-treated males, but not females, were found to have significantly less inflammation as compared to TCDD alone, but significantly higher levels of serum ALT that corresponds with greater expression of AHR-battery genes, such as *Cyp1a1*, *Cyp1a2*, and *Cyp1b1* (Table 1). Female mice co-treated with S + T, while not having differences in histologic inflammation scores, (Table 2), were found to have increased hepatic vacuolization as compared to females that received TCDD alone (Fig. 2A). Males, on the other hand, were not found to have differences in the level of vacuolization in comparing S + T co-treated and TCDD treatment groups.

Given that TCDD exposure mediates increase in hepatic lipid accumulation in mice, we hypothesized that the increase in hepatic weight and vacuolization in the S + T co-treated females was due to discrepancies in lipid accumulation. While there were no differences in serum triglyceride levels across exposures (Table 2), hepatic oil red O (ORO) staining suggests differences in neutral lipid accumulation across treatments (Fig. 2A). As seen in previous studies, ORO quantification confirmed that TCDD increased hepatic neutral lipid levels in both sexes regardless of simvastatin treatment (Fig. 2B)^26^. The quantification results were confirmed via measuring triglyceride content in hepatic lipid extract (Fig. 2C). Interestingly, livers from S + T co-treated mice in both sexes had significantly less ORO-staining as compared to TCDD alone (Fig. 2B). Quantification of triglyceride content in hepatic lipid extracts confirmed the significant difference in females, but not males (Fig. 2C).

### Impacts on hepatic lipid metabolism

Serum free fatty acids (FFA), ketone body (i.e. beta-hydroxybutyrate; BH) levels, and expression of hepatic peroxisome proliferator-activator alpha (*Ppara*) were assayed to investigate whether lipid metabolism is being utilized for energy production (Table 1; Table 2). In males, FFA and triglyceride levels in serum were unaffected by treatments. BH levels, on the other hand, were found to be significantly higher in males that received TCDD regardless of diet. In females, we observed a different pattern as the S + T co-treatment led to significantly lower levels of free fatty acids and BH levels in the serum as compared to simvastatin and TCDD alone (Table 2). Like in males, triglyceride levels in the serum were not affected in females by any treatment. The expression of *Ppara* and several PPAR-alpha target genes, such as *Cyp4a10* and *Cyp4a14*, male and female mice were found to follow a similar pattern (Table 1). Hepatic expression of *Ppara* was found to be significantly lower in both sexes treated with TCDD as compared to controls and mice that received the S + T co-treatment. Male and female mice treated with TCDD alone had significantly less expression of *Cyp4a10* and *Cyp4a14* in the liver as compared to control and S + T co-treated mice. As such, expression of PPAR-alpha target genes were observed to follow a similar pattern to *Ppara* expression suggesting that PPAR-alpha activity is also further repressed in S + T co-treated females as compared to TCDD treatment.

### Alterations in hepatic glycogen metabolism

**Figure 3:**
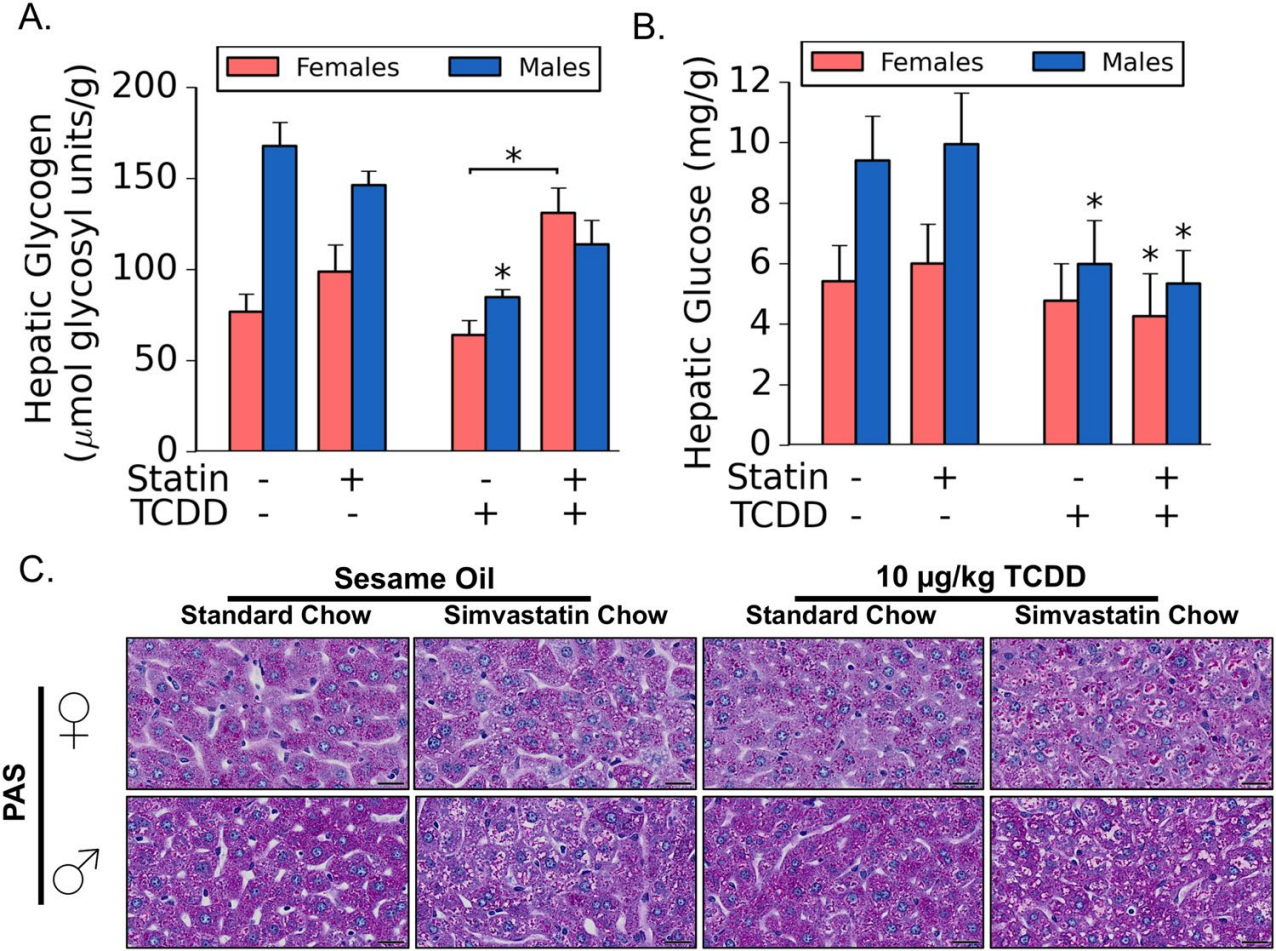
Impact of simvastatin and TCDD co-exposure on hepatic glycogen metabolism. Hepatic glycogen levels were inferred based on background hepatic glucose levels (**A**,**B**, respectively). Results are reported as fold changes relative to vehicle control (i.e. sesame oil + standard chow). Sample sizes (n) were ≥5 in all cases. Asterisks (*) indicate statistically significant differences (p ≤ 0.05) as compared to the respective vehicle control (i.e. sesame oil vs. TCDD treatment or simvastatin-treatment vs. simvastatin + TCDD co-treatment) or between means indicated by brackets. Periodic acid-Schiff stain (PAS) was used to confirm differences in glycogen levels; a representative sample was chosen for each treatment group (**C**). Scale bars represents 20 µm.

As the increased hepatic weight and histological differences in the S + T co-treated females as compared to the TCDD treatment alone were not due to lipid accumulation, we sought to analyze the hepatic glycogen levels in each group. In females, we found significantly more hepatic glycogen in the S + T co-treatment group as compared to TCDD alone (Fig. 3A). The results were confirmed with a periodic acid-Schiff (PAS) stain which detects polysaccharides such as glycogen (Fig. 3C). While TCDD treatment alone did not impact hepatic glucose in females, the S + T co-treated mice had significantly less hepatic glucose as compared simvastatin alone (Fig. 3B). As such, the glycogen:glucose ratio is >2 fold higher in S + T co-treated females as compared to TCDD-treated females

Of particular note was a difference in hepatic glycogen and glucose patterns observed in males. In the male mice, TCDD-treatment led to a significant drop in hepatic glycogen (Fig. 3A). While there was a slight increase in hepatic glycogen levels of S + T co-treated males as compared to TCDD alone, the difference was not near significance. The results were further confirmed with PAS stain (Fig. 3C). As opposed to no changes observed in hepatic glucose levels in females, male mice treated with TCDD and co-treated with S + T were observed to have significant reductions in hepatic glucose levels (Fig. 3B).

Expression of several genes important in hepatic glycogen metabolism were assessed to elucidate a potential mechanism behind the altered levels observed in females (Table 1). In females, expression of genes that are involved in glycogen anabolism, such as glycogen synthase (*Gys2*), UDP-glucose pyrophosphorylase, (*Ugp2*), and glycogen-branching enzyme (*Gbe1*) were significantly repressed in the liver following TCDD treatment. Similarly, expression of the glycogen phosphorylase gene (*Pygl*), which is involved in regulating glycogen catabolism, was significantly repressed by TCDD in females as well. Simvastatin treatment alone led to significantly less expression of *Gbe1* and *Pygl* as well as two other enzymes important to glycogen anabolism: hexokinase 1 (*Hk1*) and phosphoglucomutase 1 (*Pgm1*) in females. However, in comparing expression of these 5 genes across female S + T co-treated mice and TCDD alone, the co-treatment only induced further repression of hepatic *Gys2* in females. In males, TCDD treatment had a similar impact as observed in females on *Gys2*, *Ugp2*, *Gbe1*, and *Pygl* as these genes were found significantly repressed as compare to controls. While simvastatin alone was found to impact expression of *Gbe1*, *Pygl*, *Hk1*, and *Pgm1* in females, expression of these genes were not different amongst male treatment groups. Similarly, unlike *Gys2*, S + T co-treatment was not found to impact these genes as compared to TCDD exposure alone in male mice.

## Discussion

Previous *in vitro-* and rodent-based studies have indicated that exposures to AHR ligands repress expression of genes involved in cholesterol biosynthesis and ultimately lead to reductions of serum TC, HDL, and LDL in serum^22,23^. To characterize the impact of TCDD-induced repression of cholesterol biosynthesis in TCDD-elicited liver pathology, C57BL/6 mice were gavaged with TCDD in the presence or absence of simvastatin, a competitive inhibitor of HMG-CoA reductase. While TCDD repression of hepatic *Hmgcr* was modest at the gene and protein level at the time of sacrifice, several other key genes, including *Dhcr7*, *Sqle*, and *Cyp51*, suggest that, overall, TCDD exposure is repressing cholesterol biosynthesis in mice of both sexes. Consistent with this observation, serum TC and HDL levels were lower following exposure to TCDD which is consistent with previous reports suggesting AHR regulates cholesterol homeostasis in mice (Table 2)^23^. Unlike previous reports, LDL levels were mostly unaffected in this study which is likely due to differences in study design^23^. Simvastatin, while increasing *Hmgcr* gene expression (Fig. 1A), did not alter HMGCR protein levels or impact TC or LDL levels in serum at the time of sacrifice (Fig. 1B; Table 2). Notably, statins primarily reduce LDL levels in humans^30^. Previous studies have shown that statins are less effective in lower circulating LDL in mice since, unlike humans, HDL is the predominant lipoprotein^31^. Along these lines, simvastatin was found to have a significant impact on serum HDL in females, but not males (Table 2). As the primary role of HDL is in reverse-cholesterol transport, these results suggest that simvastatin-treated female mice down-regulate transport of cholesterol from tissues to the liver. Gene expression of the scavenger receptor B1 (*Scarb1*), which serves as the HDL receptor, was not impacted by simvastatin in the liver (Table 1). While expression of the apolipoprotein A1 gene (*Apoa1*), the primary lipoprotein component of HDL, was significantly increased in males treated with simvastatin as compared vehicle mice, *Apoa1* expression was not different in females (Table 1). APOA1 is essential for HDL formation with *Apoa1*^*−/−*^ mice exhibiting lower circulating levels of HDL^32^. Similarly, simvastatin-mediated repression of lecithin cholesterol acyltransferase (*Lcat*) was found to be near statistical significance in the females, but not males (p = 0.06; Table 1). Hepatic LCAT esterifies cholesterol to promote HDL maturation^33^. LCAT deficiency decreases HDL and, ultimately, catabolism of APOA1^34^. Given these results, we hypothesize that repression of *Apoa1* and/or down-regulation of *Lcat* at the transcriptional level may be involved in the simvastatin-mediated decrease in HDL in females.

Histologically, male mice were found to be more sensitive to TCDD-induced injury when co-treated with simvastatin. The S + T co-treatment, while apparently reducing inflammation in the males, led to an increase in ALT levels suggesting that co-treatment increases the severity of TCDD-induced liver injury (Table 2; Fig. 2A). S + T co-treated males, as compared to TCDD alone, also displayed increased AHR-mediated transcription as indicated by induction of several AHR-battery genes, such as *Cyp1a1*, *Cyp1a2*, and *Cyp1b1* (Table 1). Given that TCDD-mediated liver injury is AHR-dependent^21,35^, we hypothesize that the more severe liver damage elicited by the S + T co-treatment is due to enhanced AHR activity. Interestingly, this trend was not present in female mice. The mechanism behind the increased AHR-mediated transcription in the S + T treated male mice requires further investigation.

As seen in previous studies, TCDD increased hepatic cholesterol and lipid levels (Fig. 2; Table 2)^18^. S + T co-treatment, however, reduced hepatic cholesterol levels compared to TCDD in both sexes (Table 2). These results correlate with lower-levels of lipid accumulation in the S + T co-treated group (Fig. 2). Previous reports have associated NAFLD with higher-levels of HMGCR expression and accumulation of hepatic free cholesterol in humans^36^. Epidemiological studies have also suggested that statins protect against hepatic steatosis^37^. Collectively, these results suggest that AHR-mediated repression of *Hmgcr* and other genes involved in cholesterol biosynthesis may protect against TCDD-induced steatosis in mice. Our results support this hypothesis as simvastatin, an inhibitor of HMGCR, reduced hepatic cholesterol and lipid levels in TCDD treated mice.

The simvastatin-mediated protection against TCDD-elicited hepatic lipid deposition appears to be sex-specific (Fig. 2). The decreased serum FFA levels in S + T co-treated females suggest that S + T co-treated female mice have lower levels of mobilized lipids to deposit in the liver (Table 2). Interestingly, S + T co-treated females have significantly lower levels of hepatic *Ppara* gene expression, a master regulator of fatty acid oxidation, which coincides with decreases in PPAR-alpha target genes, such as *Cyp4a10* and *Cyp4a14* (p < 0.05; Table 1)^38^. Serum ketone bodies are also reduced in S + T co-treated females compared to the TCDD-treatment (p < 0.05; Table 2). Collectively, this suggests S + T co-treated females are likely not utilizing hepatic lipids as an energy source, but rather the simvastatin-mediated reduction hepatic lipid levels are due to less lipid mobilization to the liver. While having comparable levels of hepatic ORO staining and triglyceride levels in the liver as the females, male mice appear to be in a different metabolic state. Gene expression of *Ppara*, while not statistically significant, was slightly increased while PPAR-alpha target genes were significantly higher in S + T co-treated males as compared to TCDD alone (Table 1). The gene expression results suggest that S + T co-treated males may have less repression of fatty acid oxidation in the liver as compared to TCDD alone. While TCDD exposures are thought to repress beta-oxidation, male mice in our study had increases in the levels of ketone bodies in the serum suggesting that fatty acids are being utilized for energy production. The hepatic gene expression suggests that S + T co-treated males have more PPAR-alpha-related activity in the liver (Table 1). However, the increases in PPAR-alpha-related activity in the S + T co-treated liver of males does not appear to be severe enough to induce a systemic increase in ketone body levels in the blood (Table 2).

The most surprising phenotype was a significant increase in hepatic glycogen content found within the S + T co-treated females as compared to TCDD alone (Fig. 3A,C). The phenotype coincides with an increase in liver weight (Table 2). While not as pronounced in females, the S + T co-treated males had a similar increase in glycogen deposition and liver weight (Fig. 3A; Table 2). Previous studies report that TCDD depletes hepatic glycogen stores^20^. We hypothesize S + T co-treatment is driving a metabolic phenotype similar to a glycogen storage disease (GSD). GSDs are a diverse-set of autosomal recessively inherited metabolic disorders mediated through enzyme deficiencies^39^. As the mice were fasted prior to sacrifice, we are hypothesizing that the greater level of glycogen content in S + T co-treated females as compared to TCDD alone is due to an impairment of glycogen catabolism as opposed to increased anabolism. Repression of glycogen phosphorylase (*Pygl*) gene expression, which is the rate-limiting enzyme of glycogenolysis, supports this hypothesis (Table 1). Similarly, S + T co-treatment repressed glycogen synthase (*Gys2)* impairing glycogen anabolism and, thus, further supports this hypothesis (Table 1). However, expression of other genes involved in glycogen anabolism, such as *Ugp2* and *Gbe1*, where not different in comparing S + T co-treated and TCDD treated females. Furthermore, we do note that PYGL and GYS2 activities are primarily regulated post-translation which we did not assay^40^. As such, we hypothesize that glycogen catabolism is being altered post-translationally. Further studies are required to screen the expression and activity of enzymes involved glycogen catabolism to test this hypothesis and elucidate the mechanism behind this phenotype.

In conclusion, the novelty of this study is in the functional characterization of sex-specific roles played by HMGCR in TCDD-mediated toxicity in the liver. Results suggest that TCDD-mediated repression of cholesterol biosynthesis protects against lipid accumulation in the liver of mice, but is most prominent in female mice. Although lipid accumulation was reduced in both sexes, the S + T co-treatment increased AHR-mediated liver injury as compared to TCDD treatment alone in males mice only. On the other hand, the S + T co-treatment was found to lead to increased hepatic glycogen levels that was unique to female mice. While further research is needed to better understand the mechanism and sex-differences in mice, our results suggest that HMGCR plays a crucial role in TCDD-elicited liver toxicity. Furthermore, results from this mouse experiment suggest that individuals who take statins may be protected from AHR-mediated steatosis, but are at greater risk for TCDD-elicited liver injury and alterations in glycogen metabolism in a sex-specific manner. Further research is required to establish whether the findings are relevant to the human population.

## Supplementary information

Supplemental information and data
