# Supplementary material for: Characterizing the Role of HMG-CoA Reductase in Aryl Hydrocarbon Receptor-Mediated Liver Injury in C57BL/6 Mice
Source: Sci Rep. 2019 Nov 1;9:15828. doi: 10.1038/s41598-019-52001-2 (PMC6825130; doi:10.1038/s41598-019-52001-2)
Supplement: Supplementary file 1 — Supplemental information and data [file 41598_2019_52001_MOESM1_ESM.docx]

**Title:** Characterizing the Role of HMG-CoA Reductase in AHR-Mediated Liver Injury in C57BL6 Mice

**Authors:** Peter Dornbos^1,2^, Amanda Jurgelewicz^2,3^, Kelly A. Fader^1,2^, Kurt Williams^4^, Timothy R. Zacharewski^1,2^, John J. LaPres^1,2^

**Affiliations:** ^1^Department of Biochemistry and Molecular Biology, Michigan State University, East Lansing, MI 48824, ^2^Institute for Integrative Toxicology, Michigan State University, East Lansing, MI 48824, ^3^Department of Pharmacology and Toxicology, ^4^Department of Pathobiology and Diagnostic Investigation, Michigan State, East Lansing, MI 48824

**Corresponding Author:** Dr. John J. LaPres

**Address:** Michigan State University, 603 Wilson Road, Room 224, East Lansing, MI 48824-1319, USA

**Email:** lapres@cns.msu.edu

**Telephone:** 517-432-

**Supplemental Tables**

**Table S1. Primer Sequences used for SYBR green-based QRTPCR.**

| **Gene** | **Official Gene ID** | **Forward** | **Reverse** |
| --- | --- | --- | --- |
| *Actb* | 11461 | TGTTACCAACTGGGACGACA | GGGGTGTTGAAGGTCTCAAA |
| *Apoa1* | 11806 | GTGGCTCTGGTCTTCCTGAC | ACGGTTGAACCCAGAGTGTC |
| *Cyp1a1* | 13076 | AAGTGCAGATGCGGTCTTCT | AAAGTAGGAGGCAGGCACAA |
| *Cyp1a2* | 13077 | CACTAACGGCAAGAGCATGA | AGCTTGCTGACGAGATGGTT |
| *Cyp1b1* | 13078 | TGCTTTTGTTTCTGCCACAG | GGGGCATGAATTCTTGTGAT |
| *Cyp4a10* | 13117 | ACCACAATGTGCATCAAGGA | CTGAGAAGGGCAGGAATGAG |
| *Cyp4a14* | 13119 | ACCTGTCACCTTCCCAGATG | AGCAAACTGTTTCCCAATGC |
| Cyp51 | 13121 | TTGAGAATTTGAGGCCAACC | CTGGATCTCATGGAGGCATT |
| Dhcr7 | 13360 | CGCTCCCAAAGTCAAGAGTC | GTGTCTTGGCCCAAATGTCT |
| Fbp1 | 14121 | GTCTGTTTCGATCCCCTTGA | TCCAGCATGAAGCAGTTGAC |
| Gbe1 | 74185 | CCATTATGCCAGAAGGCAGT | GCTTGGGTGGAAGTTGAAAA |
| *Gusb* | 110006 | GAGGATCAACAGTGCCCATT | AGGTAAGGCCACCAGAGGTT |
| *Gys2* | 232493 | GGGACACTGTGCATTGTTTG | CCGATTCGTCTAATGGTGCT |
| Hk1 | 15275 | GAGGCATCTTCGAGACCAAG | TCTCGGATCTTTTCCACCAC |
| *Hmgcr* | 15357 | GAATTGAACTCCCCATCGAG | GGATATGCTTGGCATTGACC |
| *Hprt* | 15452 | GCTTACCTCACTGCTTTCCG | ATCGCTAATCACGACGCTGG |
| *Lcat* | 16816 | GCTCCTCAATGTGCTCTTCC | AATCCAGCCAGATGGTGAAG |
| *Ldlr* | 16835 | TCCTGGAGATGTGATGGACA | GAGCCATCTAGGCAATCTCG |
| Pck1 | 18534 | CTGGCACCTCAGTGAAGACA | TCGATGCCTTCCCAGTAAAC |
| Pgm1 | 66681 | TCAGGCCATTGAGGAAAATC | CGAACTTCACCTTGCTCTCC |
| *Ppara* | 19013 | GAGGGTTGAGCTCAGTCAGG | GGTCACCTACGAGTGGCATT |
| *Pygl* | 110095 | ACCAAATCGACAATGGCTTC | CCATTGTGTTCCAGGCTTTT |
| Sqle | 20775 | CCTGTTGGGTTGCTTTCAAT | CACGTGGACTCCCTTTCAAT |
| Ugp2 | 216558 | TCTGGCTTGCTCGATACCTT | GAGCGATTTCCACCAGTCTC |

**Table S2.HMGCR densitometry raw data.** Arbitrary and normalized densitometry units are reported for HMGCR western blots. ACTB values were normalized to the darkest ACTB band (Relative NC) on each respective gel and HMGCR values are normalized to Relative NC values.

|  |  |  |  |  |  |  |  |  |  |  |  |  |
| --- | --- | --- | --- | --- | --- | --- | --- | --- | --- | --- | --- | --- |
|  |  |  | Female | | | | | Male | | | | |
|  | **Gel ID** | **Treatment** | **Sample ID** | **Hmgcr** | **B-actin** | **Relative NC** | **Normalized Protein** | **Sample ID** | **Hmgcr** | **B-actin** | **Relative NC** | **Normalized Protein** |
|  | Gel 1 | Vehicle | 3 | 427000 | 146000 | 0.69 | 620027.40 | 33 | 369000 | 150000 | 0.71 | 521520.00 |
|  |  | TCDD | 9 | 360000 | 182000 | 0.86 | 419340.66 | 43 | 333000 | 197000 | 0.93 | 358355.33 |
|  |  | Vehicle + Simvastatin | 22 | 397000 | 184000 | 0.87 | 457413.04 | 52 | 278000 | 97300 | 0.46 | 605714.29 |
|  |  | TCDD + Simvastatin | 27 | 374000 | 212000 | 1.00 | 374000.00 | 57 | 225000 | 138000 | 0.65 | 345652.17 |
|  | Gel 2 | Vehicle | 7 | 284000 | 138000 | 0.56 | 508318.84 | 35 | 268000 | 149000 | 0.60 | 444268.46 |
|  |  | TCDD | 11 | 274000 | 170000 | 0.69 | 398105.88 | 47 | 260000 | 247000 | 1.00 | 260000.00 |
|  |  | Vehicle + Simvastatin | 20 | 270000 | 157000 | 0.64 | 424777.07 | 51 | 264000 | 144000 | 0.58 | 452833.33 |
|  |  | TCDD + Simvastatin | 28 | 251000 | 213000 | 0.86 | 291065.73 | 58 | 274000 | 206000 | 0.83 | 328533.98 |
|  | Gel 3 | Vehicle | 8 | 300000 | 101000 | 0.64 | 469306.93 | 34 | 253000 | 66700 | 0.42 | 599310.34 |
|  |  | TCDD | 10 | 240000 | 116000 | 0.73 | 326896.55 | 45 | 277000 | 123000 | 0.78 | 355821.14 |
|  |  | Vehicle + Simvastatin | 23 | 334000 | 78600 | 0.50 | 671399.49 | 54 | 237000 | 91200 | 0.58 | 410592.11 |
|  |  | TCDD + Simvastatin | 32 | 207000 | 98700 | 0.62 | 331367.78 | 59 | 304000 | 158000 | 1.00 | 304000.00 |
|  | Gel 4 | Vehicle | 5 | 344000 | 214000 | 0.64 | 535289.72 | 39 | 343000 | 188000 | 0.56 | 607547.87 |
|  |  | TCDD | 14 | 288000 | 213000 | 0.64 | 450253.52 | 45 | 368000 | 333000 | 1.00 | 368000.00 |
|  |  | Vehicle + Simvastatin | 24 | 390000 | 206000 | 0.62 | 630436.89 | 53 | 367000 | 248000 | 0.74 | 492786.29 |
|  |  | TCDD + Simvastatin | 29 | 371000 | 253000 | 0.76 | 488312.25 | 62 | 299000 | 272000 | 0.82 | 366055.15 |
|  | Gel 5 | Vehicle | NA | NA | NA | NA | NA | 38 | 284000 | 132000 | 0.58 | 486242.42 |
|  |  | TCDD | NA | NA | NA | NA | NA | 41 | 255000 | 195000 | 0.86 | 295538.46 |
|  |  | Vehicle + Simvastatin | NA | NA | NA | NA | NA | 50 | 263000 | 106000 | 0.47 | 560735.85 |
|  |  | TCDD + Simvastatin | NA | NA | NA | NA | NA | 60 | 311000 | 226000 | 1.00 | 311000.00 |
|  | Gel 6 | Vehicle | 4 | 345000 | 168000 | 0.90 | 384017.86 | NA | NA | NA | NA | NA |
|  |  | TCDD | 13 | 262000 | 148000 | 0.79 | 331040.54 | NA | NA | NA | NA | NA |
|  |  | Vehicle + Simvastatin | 18 | 411000 | 137000 | 0.73 | 561000.00 | NA | NA | NA | NA | NA |
|  |  | TCDD + Simvastatin | 25 | 548000 | 187000 | 1.00 | 548000.00 | NA | NA | NA | NA | NA |

**Supplemental Figures**

**Figure S1. Full gel image associated with cropped blot pictured Figure 1C.**

**
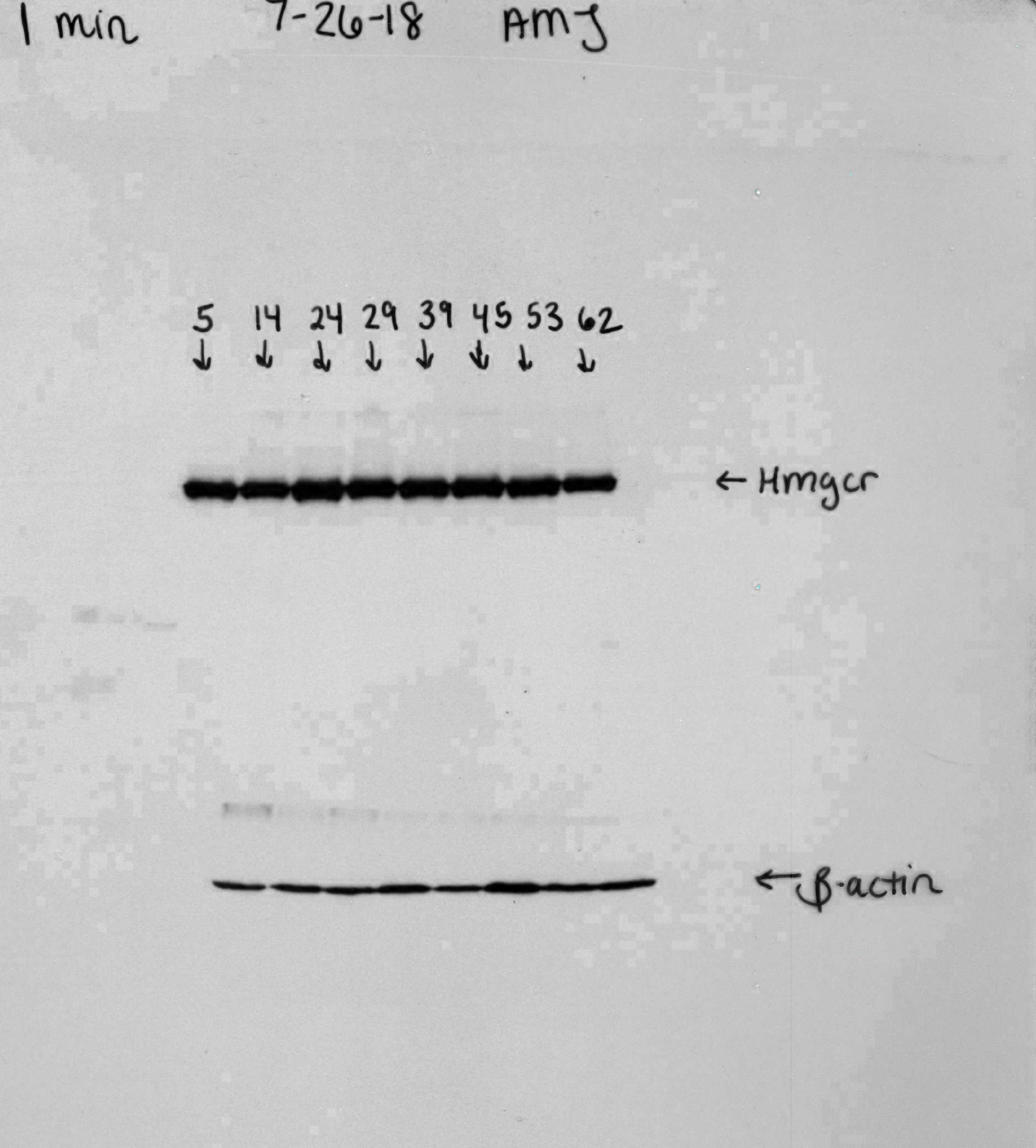
**
